# Structure of MltG from *Mycobacterium abscessus* reveals structural plasticity between composed domains

**DOI:** 10.1107/S2052252524008443

**Published:** 2024-11-01

**Authors:** Gwan Hee Lee, Subin Kim, Do Yeon Kim, Ju Hee Han, So Yeon Lee, Jun Hyuck Lee, Chang Sup Lee, Hyun Ho Park

**Affiliations:** a College of Pharmacy Chung-Ang University Seoul06974 Republic of Korea; b Department of Global Innovative Drugs Graduate School of Chung-Ang University Seoul06974 Republic of Korea; c Unit of Research for Practical Application, Korea Polar Research Institute, Incheon21990, Republic of Korea; d Department of Polar Sciences University of Science and Technology Incheon21990 Republic of Korea; e College of Pharmacy and Research Institute of Pharmaceutical Science, Gyeongsang National University, Jinju52828, Republic of Korea; University of Michigan, USA

**Keywords:** crystal structures, lytic transglycosylase, MltG, *Mycobacterium abscessus*, protein structures, X-ray crystallography, antibiotic resistance, structural plasticity

## Abstract

MltG, a membrane-bound lytic transglycosyl­ase, has roles in terminating glycan polymerization in peptidoglycan and incorporating glycan chains into the cell wall, making it significant in bacterial cell-wall biosynthesis and remodeling. This study provides the first reported MltG structure from *Mycobacterium abscessus* (maMltG), a superbug that has high antibiotic resistance. Our structural and biochemical analyses revealed that MltG has a flexible peptidoglycan-binding domain and exists as a monomer in solution. Further, the putative active site of maMltG was disclosed using structural analysis and sequence comparison. Overall, this study contributes to our understanding of the transglycosyl­ation reaction of the MltG family, aiding the design of next-generation antibiotics targeting *M. abscessus.*

## Abstract 2

We provide the structure of MltG of the lytic transglycosyl­ase family in this study. We show that MltG has a flexible peptidoglycan-binding domain and exists as a monomer in solution. Further, the putative active site of *Mycobacterium abscessus* MltG has been revealed using structural analysis and sequence comparison. This research significantly advances our comprehension of the transglycosyl­ation process mediated by the MltG family, providing valuable insights that can inform the development of next-generation antibiotics to specifically target *M. abscessus*.

## 1. Introduction

**Figure 1:**
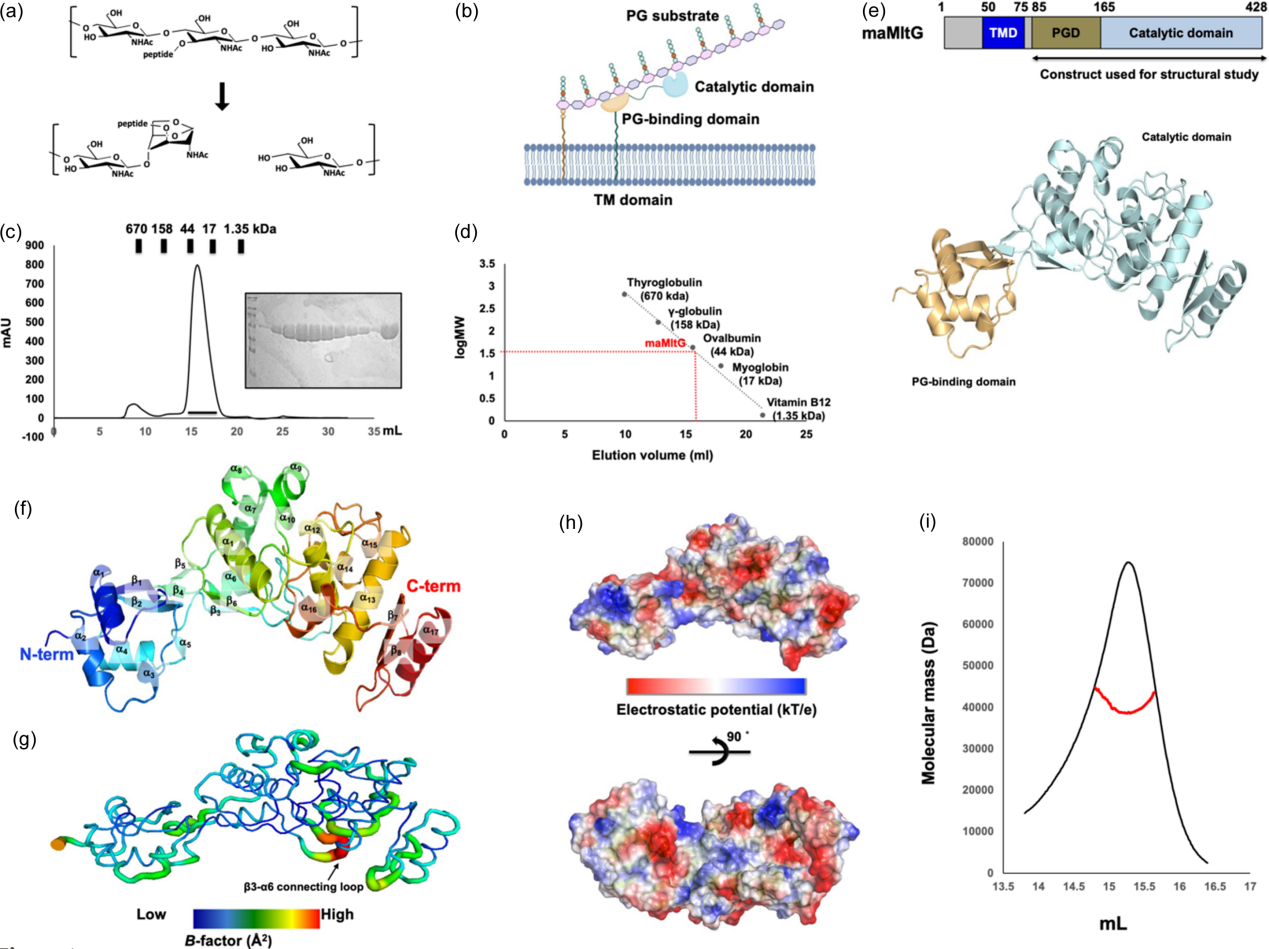
Crystal structure of MltG from *M. abscessus* (maMltG). (*a*) Schematic showing the reaction of LT. (*b*) Cartoon showing the domain boundary of MltG. (*c*) SEC profiles for purifying maMltG. Eluted standard-size markers are above the profile. The SDS–PAGE loaded with main peak fractions is shown on the right side of the main peak. The black line under the main peak indicates the loaded fractions for SDS–PAGE. (*d*) Elution volume line fitting in SEC versus the size marker and maMltG molecular weight logarithm. The red point on the fitting line indicates the elution volume. Size marker molecular weights are indicated in the standard line. (*e*) Domain composition and structure of maMltG. (*f*) Ribbon representation of maMltG. The rainbow color scheme was used for tracing the N- to C-terminus. Helices and sheets are labeled α and β, respectively. (*g*) Putty representation conveying the *B* factor distribution. Relative *B* factor values are visualized using a rainbow spectrum from red to violet. (*h*) Electrostatic surface representation. The scale ranges from −6.2 kT/e (red) to +6.2 kT/e (blue). (*i*) MALS profile derived from the primary SEC peak. Experimental MALS data (red line) are plotted as SEC elution volume (*x* axis) versus absolute molecular mass (*y* axis) distributions on the SEC chromatogram (black) at 280 nm.

The bacterial cell membrane is surrounded by a peptidoglycan (PG) layer known as the sacculus which prevents the cell from bursting and helps maintain its shape (Egan *et al.*, 2020 ▸). PG is a unique bacterial heteropolymer synthesized from long glycan chains of β-1,4-linked *N*-acetyl­muramic acid (MurNAc) and *N*-acetyl­glucosamine (GlcNAc) sugars connected by short-stem peptides, forming a net-like macromolecule surrounding the cell (Egan *et al.*, 2015 ▸; Kussau *et al.*, 2020 ▸). Two essential enzymes, hydro­lases and synthases, called high-molecular-weight penicillin-binding proteins (PBPs), are required for PG synthesis (Yunck *et al.*, 2016 ▸). Polymerization by synthases results in the anchoring of a glycan chain to the membrane (Bohrhunter *et al.*, 2021 ▸; Taguchi *et al.*, 2021 ▸). One type of enzyme involved in this process is membrane-bound glycosidase, which cleaves the PG sugar backbone (Taguchi *et al.*, 2021 ▸). Lytic transglycosyl­ase (LT), anchored to the cytoplasmic membrane, is a representative glycosidase enzyme that catalyzes nonhydrolytic cleavage of the β-1,4 glycosidic bond between MurNAc and GlcNAc to produce muropeptide products containing a 1,6-anhydro-MurNAc (anhMurNAc) end [Fig. 1 ▸(*a*)] (Taguchi *et al.*, 2021 ▸). This transglycosyl­ation reaction is a vital step in PG metabolism, because it enables the integration of newly synthesized PG into the cell wall by releasing glycan strands anchored to the cell membrane (Taguchi *et al.*, 2021 ▸; Dik *et al.*, 2017 ▸). MltG is a member of the LT family, which includes seven lipoproteins anchored to the outer membrane (MltA, MltB, MltC, MltD, MltE, MltF and RIpA) and a soluble periplasmic enzyme (Slt) (Sassine *et al.*, 2021 ▸). MltG is a plasma membrane enzyme conserved in other bacterial species such as *Escherichia coli*, *Bacillus subtilis*, *Pseudomonas aeruginosa*, *Streptococcus pneumoniae* and *Mycobacterium abscessus* (Sassine *et al.*, 2021 ▸; Lee *et al.*, 2017 ▸). In a previous study, MltG was characterized as the terminase responsible for achieving PG integration into the cell wall through cleavage of the newly synthesized glycan strand (Bohrhunter *et al.*, 2021 ▸; Sassine *et al.*, 2021 ▸). *E. coli* MltG comprises three domains: an N-terminal transmembrane domain (TMD), a predicted PG-binding domain and a C-terminal YceG (MltG) catalytic domain (Bohrhunter *et al.*, 2021 ▸). The N-terminal TMD of MltG is not required for its function, but is essential for accessing the membrane (Bohrhunter *et al.*, 2021 ▸). Meanwhile, the PG-binding domain is essential for effective PG binding (Bohrhunter *et al.*, 2021 ▸).

Infections caused by non-tuberculous mycobacteria (NTM) are increasing worldwide and present a formidable challenge for treatment because of their inherent resistance to numerous common antibiotics (Johansen *et al.*, 2020 ▸). Among the rapidly growing NTM, *Mycobacterium abscessus* is a pathogenic gram-positive bacterium found in various environments, including common soil and water contaminants. It is an opportunistic pathogen that causes human infections, especially in individuals with weakened immune systems, such as those with cystic fibrosis or various other chronic lung diseases (Luthra *et al.*, 2018 ▸; Johansen *et al.*, 2020 ▸). This bacterium has a very high inherent resistance to most antibiotics commonly used for gram-negative and gram-positive bacterial infections, and is classified as a superbug (Luthra *et al.*, 2018 ▸; Hashemi Shahraki & Mirsaeidi, 2021 ▸). Therefore, significant emphasis has been placed on developing novel antibiotics and vaccines targeting *M. abscessus* (Butler *et al.*, 2023 ▸; Williams, 2007 ▸).

In gram-positive bacteria, the three PG layers that make up the cell wall result in low antibiotic permeability and thera­peutic efficacy (Sayed *et al.*, 2020 ▸; Jarlier & Nikaido, 1994 ▸). The low permeability of mycobacterial cell walls to antibiotics is a major factor in antibiotic resistance (Jarlier & Nikaido, 1994 ▸). As transglycosyl­ation is essential for PG synthesis and bacterial survival, LT is a major target for the development of new antibiotics. Therefore, identifying the structure and function of bacterial LT, including the MltG family, could offer insights into bacterial metabolism and pathogenesis, ultimately contributing to the development of effective antibiotics. Therefore, in this study, we determined the structure of the *M. abscessus*MltG (*maMltG*). We found that maMltG is a monomer in solution and revealed its active site based on structure and sequence conservation analyses. We also examined the structural plasticity between composed domains of the MltG family. Overall, the results of this study will contribute to broader efforts aimed at combating antibiotic resistance and addressing the urgent need for new antibiotics to treat infections caused by multidrug-resistant pathogens, such as *M. abscessus*.

## 2. Materials and methods

### 2.1. Protein expression and purification

The N-terminal-truncated maMltG gene, containing amino acids 85–428 was synthesized by BIONICS (Dae-Joen, Republic of Korea). The sequence information for the full-length MltG gene was obtained from GenBank (accession No. WP_302359996). A pET28a vector that had been digested at NdeI and XhoI restriction sites was used to construct the expression plasmid by inserting the synthesized gene product. The maMltG expression plasmid was transformed into *E. coli* strain BL21(DE3). A selected single colony was cultured overnight at 37°C in 10 ml of lysogeny broth including 50 µg ml^−1^ kanamycin. Next, 1 l of the medium was inoculated using this culture. Overexpression of the target protein was induced by adding 0.25 m*M* of iso­propyl β-d-1-thio­galactopyran­oside to the solution when the optical density at 600 nm reached approximately 0.6–0.8. Subsequently, the cells were cultured overnight at 20°C in a shaking incubator. The bacterial cells were then harvested by centrifugation at 3500*g* for 15 min at 20°C. The resulting cell pellet was resuspended in 10 ml of lysis buffer composed of 20 m*M* Tris–HCl (pH 8.0) and 500 m*M* NaCl. The cells were disrupted on ice using a sonicator after adding 20 m*M* phenyl­methyl­sulfonyl fluoride (Sigma–Aldrich, St Louis, USA). The cell lysate was subjected to centrifugation at 10 000*g* for 30 min at 4°C.

The supernatant obtained after separation was gently stirred with a nickel nitrilo­tri­acetic acid resin (QIAGEN, Hilden, Germany) for two hours at 4°C. Subsequently, the supernatant/Ni-NTA resin mixture was transferred to a gravity flow column and washed with 25 ml of washing buffer [20 m*M* Tris–HCl (pH 8.0), 500 m*M* NaCl and 25 m*M* imidazole]. The column was loaded with 650 ml of elution buffer [20 m*M* Tris–HCl (pH 8.0), 500 m*M* NaCl and 250 m*M* imidazole] to extract bound proteins. The resultant eluate was subjected to further purification using size-exclusion chromatography (SEC) on an ÄKTA Explorer system (GE Healthcare, Chicago, IL, USA). For this, a 24 ml Superdex 200 Increase 10/300GL column (GE Healthcare, Chicago, IL, USA) pre-equilibrated with SEC buffer [20 m*M* Tris–HCl (pH 8.0) and 150 m*M* NaCl] was used. The peak fractions were collected and concentrated to 6 mg ml^−1^. The concentrated proteins were then flash-frozen in liquid nitro­gen and stored at −80°C until further use. Protein purity was analyzed using SDS–PAGE.

### 2.2. Crystallization and data collection

The hanging-drop vapor diffusion method was used to crystallize the maMltG protein. The reservoir (1 µl) and protein (1 µl) solutions were combined first, and the combined droplet was then allowed to equilibrate with 300 ml of mother liquor at 20°C. The initial crystal was obtained using a reservoir solution comprising 0.1 *M* Tris–HCl (pH 7.4), 1 *M* sodium citrate and 0.2 *M* NaCl. High-quality crystals were obtained from a reservoir solution comprising 0.1 *M* Tris–HCl (pH 7.4), 0.9 *M* sodium citrate, 0.2 *M* NaCl and 40%(*v*/*v*) acetone, following further adjustment of the initial crystallization conditions. The crystals appeared after 15 days and attained a maximum size of 0.1 × 0.2 × 0.2 mm. The crystals were mounted and rapidly frozen in a nitro­gen stream at −178°C after immersion in the mother liquor supplemented with 20%(*v*/*v*) glycerol as a cryoprotectant for data collection. The X-ray diffraction data were collected from the 5C beamline at the Pohang Accelerator Laboratory (PAL) (Pohang, Republic of Korea). The diffraction data were indexed, integrated and scaled using the *HKL2000* program (Otwinowski & Minor, 1997 ▸).

### 2.3. Determination and analysis of the structure

The molecular replacement (MR) phasing method was employed to determine the protein structure using the *Phaser* program in the *Phenix* package (McCoy, 2007 ▸). The MR search model utilized the structural model predicted by *AlphaFold*2. The model was built and refined using the *Coot* (Emsley & Cowtan, 2004 ▸) and *phenix.refine* tools from the *Phenix* package (Adams *et al.*, 2010 ▸). The quality of the model was validated using *MolProbity* (Chen *et al.*, 2010 ▸). All structural representations were created using *PyMOL* (DeLano & Lam, 2005 ▸).

### 2.4. SEC–multi-angle light scattering analysis

Multi-angle light scattering (MALS) was used to measure the absolute molar mass of maMltG in solution. The target protein was filtered using a 0.2 µm syringe filter and loaded onto a Superdex 200 10/300 gel filtration column (GE Healthcare) pre-equilibrated in a buffer comprising 20 m*M* Tris–HCl (pH 8.0) and 150 m*M* NaCl. SEC–MALS was performed at room temperature, and the flow rate was kept at 0.4 ml min^−1^. A DAWN-TREOS MALS detector (Wyatt Technology, Santa Barbara, CA, USA) connected to the ÄKTA explorer system (GE Healthcare) detected scattered light, and the *ASTRA* software was used to analyze the results for absolute molecular mass (Wyatt Technology).

### 2.5. Sequence alignment

The amino acid sequences of MltG derived from various species were analyzed using *Clustal Omega* (https://www.ebi.ac.uk/Tools/msa/clustalo/).

## 3. Results and discussion

### 3.1. Overall structure of maMltG

MltG consists of three domains: an N-terminal TMD, a predicted PG-binding domain and a C-terminal YceG (MltG) catalytic domain (Bohrhunter *et al.*, 2021 ▸). The TMD is responsible for anchoring MltG to the cytoplasmic membrane, while the PG-binding domain is essential for PG binding [Fig. 1 ▸(*b*)] (Bohrhunter *et al.*, 2021 ▸). To obtain a soluble protein for studying the structure of maMltG, we constructed an N-terminal truncated version of the MltG expression vector, in which the N-terminal 84 residues of MltG (*i.e.* the TMD) were removed. The maMltG protein (residues 85–428, molecular weight 40.7 kDa) was purified using a rapid two-step chromatography process involving affinity chromatography and SEC. In the SEC column, maMltG was eluted at roughly 15.5 ml between ovalbumin (44 kDa) and myoglobin (17 kDa), indicating that maMltG exists as a monomer in solution [Figs. 1 ▸(*c*) and 1 ▸(*d*)]. The purified maMltG protein was crystallized and the 2.2 Å high-resolution crystal structure of maMltG was solved using the MR phasing method. The structure predicted by *Alphafold*2 was utilized as a search model for MR. The final structural model was refined to *R*_work_ = 18.15% and *R*_free_ = 21.93%, respectively. A summary of the data analysis and refinement statistics is presented in Table 1 ▸.

The crystal structure of maMltG comprises 8 β-sheets and 17 α-helices [Figs. 1 ▸(*e*) and 1 ▸(*f*)]. The catalytic domain is located in a region consisting of α12–α17 and β7–β8 [Figs. 1 ▸(*e*) and 1 ▸(*f*)]. The putative PG-binding domain consists of α1–α2 and β1–β5 [Figs. 1 ▸(*e*) and 1 ▸(*f*)]. A single maMltG molecule was identified in the asymmetric crystallographic unit. The final structural model contains the maMltG sequence from residues 85–428.

Analysis of the *B* factors reveals that the β3–α6 connecting loop has a higher *B* factor (average 62.2 Å^2^) compared with that of the other regions of the molecule (average 38.4 Å^2^) [Fig. 1 ▸(*g*)]. Additionally, electrostatic surface characterization shows that the surface comprises both positive and negative charges, along with several neutral regions [Fig. 1 ▸(*h*)].

Because a potential maMltG monomer form was derived from the SEC experiment, we analyzed the exact stoichiometry of maMltG by determining the absolute molecular weight of the purified protein in solution using a MALS experiment. The MALS experimental results showed that the absolute molecular weight of maMltG in the solution was 40.260 Da (2.7% fitted error) and the polydispersity value was 1.002 [Fig. 1 ▸(*i*)]. This value was almost identical to the theor­etical molecular weight of maMltG with a C-terminal histidine tag. Thus, we believe that maMltG exists as a monomer in solution based on the SEC and MALS results.

### 3.2. Comparison of structural differences between maMltG and its isoforms from different species

**Figure 2:**
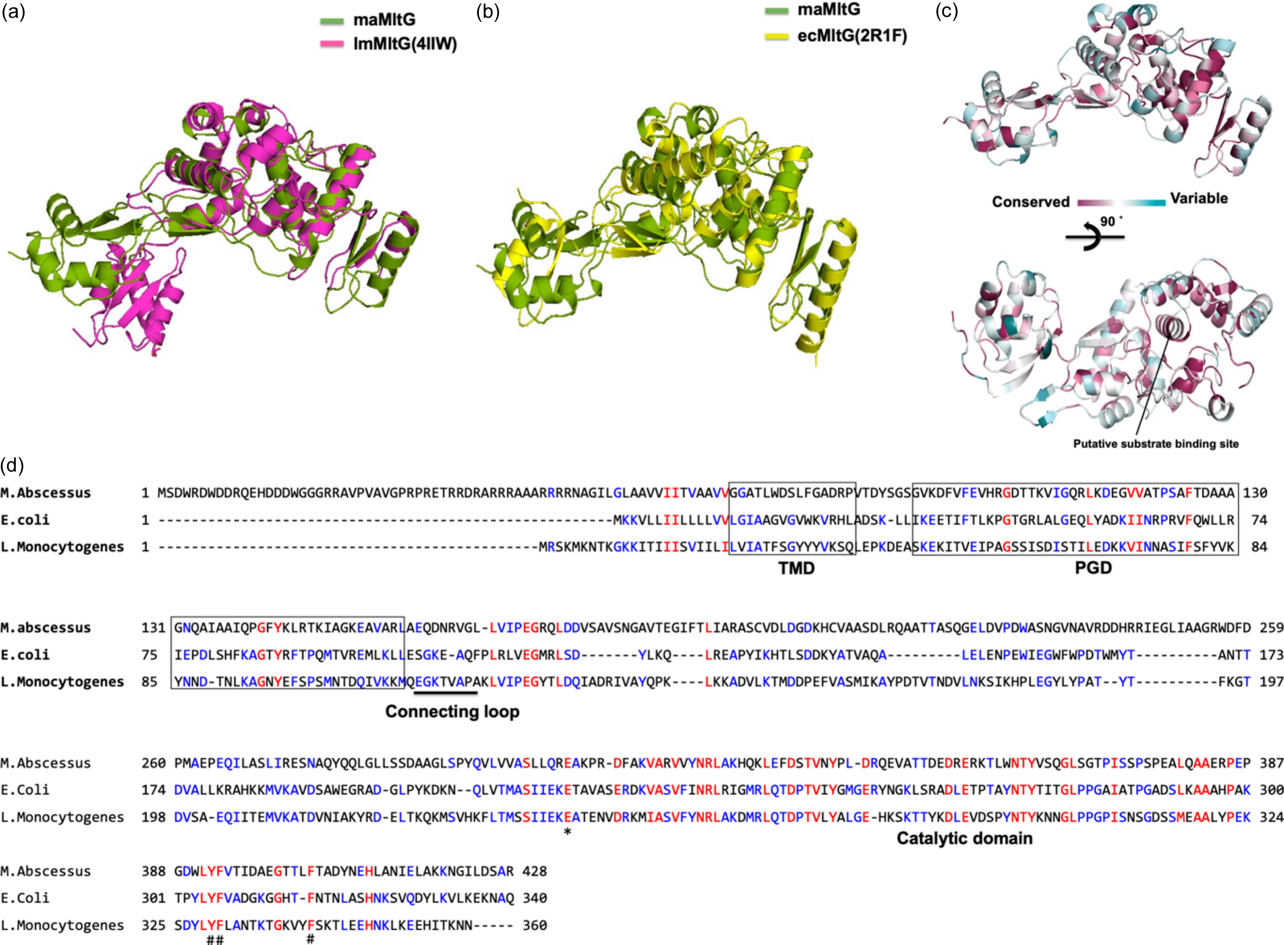
Structural comparison between maMltG and its isoforms from different species. (*a*) Pairwise structural superimposition of abYdjH (green) with (*a*) lmMltG (magenta; PDB entry 4iiw) and (*b*) ecMltG (yellow; PDB entry 2r1f). (*c*) Graphic representation of maMltG colored relative to the amino-acid sequence conservation degree generated by the *ConSurf* server. (*d*) Sequence alignment with structural homologs from different species. Residue Glu307, expected to play a crucial role in LT function as a nucleophile, is indicated by an asterisk (*). Residues that might be involved in the formation of the putative substrate binding site in the catalytic domain are indicated by a hash (#). Completely and partially conserved residues are shown in red and blue, respectively.

LTs, including MltG, play crucial roles in bacterial cell wall function and reproduction, making them promising targets for the development of novel antibiotics (Lee *et al.*, 2017 ▸; Blackburn & Clarke, 2001 ▸; Scheurwater *et al.*, 2008 ▸). Numerous studies have thus explored the structural and functional aspects of LTs (Yunck *et al.*, 2016 ▸; Bateman & Bycroft, 2000 ▸; Li *et al.*, 2012 ▸; Jing *et al.*, 2012 ▸; Kitaoku *et al.*, 2019 ▸). However, a search for structural homology using the *DALI* server (Holm & Sander, 1995 ▸) identified only two structures as structural homologs of the maMltG: YceG-like protein from *Listeria monocytogenes* (lmMltG; PDB entry 4iiw; Mibnasov *et al.*, to be published) and amino­deoxy­chorismate lyase from *E. coli* (ecMltG; PDB entry 2r1f; Patskovsky *et al.*, to be published) (Table 2 ▸). These two structures have not yet been published, although they have been deposited in the Protein Data Bank. As YceG is another name for MltG, we named the YceG-like protein from *L. monocytogenes* lmMltG and the amino­deoxy­chorismate lyase from *E. coli* as ecMltG. maMltG and other structural homologs exhibited a similar overall structure with a canonical YceG (MltG) fold containing the PG-binding domain and catalytic domain, despite their low sequence similarity (approximately 22–25%) [Figs. 2 ▸(*a*) and 2 ▸(*b*)]. However, the pairwise structural alignments showed that the root mean square deviation (RMSD) values were very high at 9.8 Å with lmMltG (when 343 residues of maMltG were aligned with 308 residues of lmMltG) and 4.1 Å with ecMltG (when 343 residues of maMltG were aligned with 259 residues of ecMltG) [Table 2 ▸ and Figs. 2 ▸(*a*) and 2 ▸(*b*)]. The high RMSD was attributed to the unmatched location of the PG-binding domain. A comparison between maMltG and other structural homologs reveals that, while the catalytic domain is quite similar, the PG-binding domain exhibits distinct structural differences by dislocating the position. For example, the PG-binding domain of lmMltG was more biased towards the catalytic domain than the PG domain of maMltG [Fig. 2 ▸(*a*)]. Moreover, the PG-binding domain of ecMltG is rotated and located far from the catalytic site compared with the PG-domain of maMltG [Fig. 2 ▸(*b*)]. This structural comparison reveals that the structure of maMltG exhibits the closest similarity to the structures of lmMltG and ecMltG. However, there is a huge structural discrepancy in the various locations of the PG-binding domain, indicating structural plasticity between the two domains of the MltG family. Since the PG-binding and catalytic domains are joined by a flexible loop, the PG-binding domain may be localized to different areas within the protein.

The catalytic domain of maMltG binds GlcNAc and plays an important role in the cleavage reaction (Jing *et al.*, 2012 ▸). Therefore, we used the *Consurf* server, which can reveal amino acid conservation to identify residues that may be involved in substrate binding (Yariv *et al.*, 2023 ▸). The results of the *Consurf* analysis show that the residues around the putative substrate-binding site are the most prominently conserved in evolutionary terms [Fig. 2 ▸(*c*)].

### 3.3. Prediction of the putative active site of maMltG

**Figure 3:**
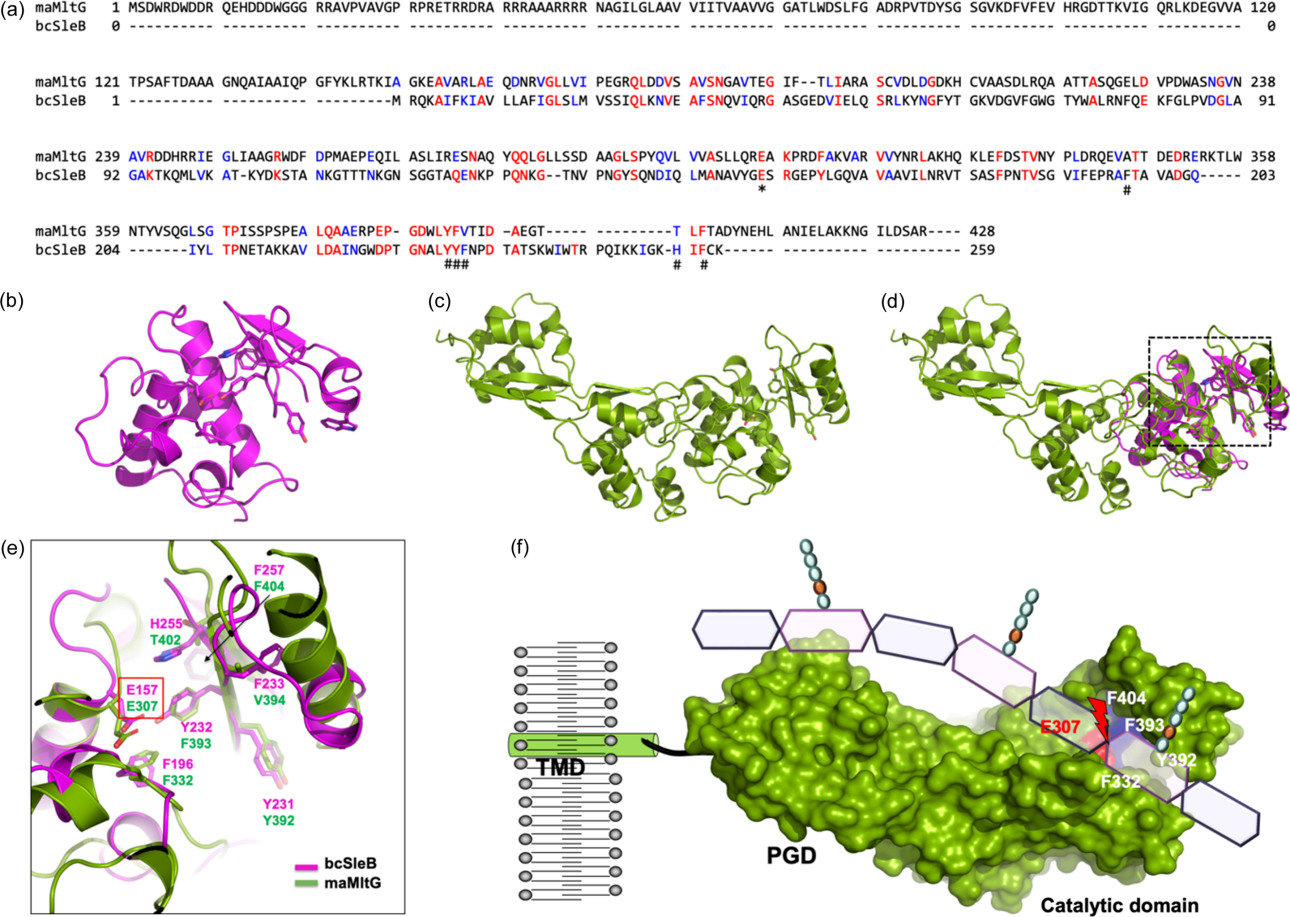
Final structural model of full-length MltG. (*a*) Sequence alignment with a member of the SleB family. Residue Glu307, expected to play a crucial role in LT function as a nucleophile, is indicated by an asterisk (*). Residues that might be involved in the formation of the putative substrate binding site in the catalytic domain are indicated by a hash (#). Completely and partially conserved residues are indicated by red and blue, respectively. (*b*) Structure of scSleB (PDB entry 4f55; Li *et al.*, 2012 ▸). (*c*) Structure of maMltG. (*d*) Pairwise structural superimposition of scSleB (magenta) with maMltG (green). Magnified region that was used for generating the panel (*e*) is indicated by dotted black square. (*e*) Close-up view of the putative active site of maMltG. The residues that form the active site in bcSleB and the corresponding residues in maMltG are labeled. (*f*) Final structural model of full-length MltG.

SleB has been extensively studied as the best-characterized enzyme in LT enzymatic activity (Li *et al.*, 2012 ▸). To identify and analyze the active site of maMltG, we compared the sequence and structure of SleB protein from Bacillus cereus (bcSleB) with that of maMltG. In this analysis, we confirmed the conservation of residue Glu307 in maMltG corresponding to Glu157 in bcSleB, which is known to act as a nucleophile for LT activity [Fig. 3 ▸(*a*)]. Additionally, residues Phe196, Tyr231, Tyr232, Phe233 and Phe257 on bcSleB, which are expected to interact with substrates, were found to be well conserved in maMltG as Phe332, Tyr392, Phe393, Val394 and Phe404, respectively [Fig. 3 ▸(*a*)]. Through sequence analysis and comparison, we confirmed that the amino acid residues involved in substrate binding, which have been studied in the other LT family members, are almost completely conserved in MltG from different species [Fig. 2 ▸(*d*)]. The analysis of the sequence and the structure revealed that the Glu307, Tyr392, Phe393 and Phe404 residues form deep grooves, constituting the putative substrate binding site in the maMltG [Fig. 2 ▸(*c*) and 2 ▸(*d*)]. Furthermore, while the structures of scSleB and maMltG appeared quite different [Figs. 3 ▸(*b*) and 3 ▸(*c*)], superimposing the structures revealed that the active site of scSleB overlapped well with the predicted active site of maMltG [Fig. 3 ▸(*d*) and 3 ▸(*e*)]. Based on these findings, we infer that the active site of maMltG forms a hydro­phobic pocket comprising residues Phe332, Tyr392, Phe393, Val394 and Phe404, facilitating the binding of PG substrate, while Glu307 acts as the catalytic residue for LT activity, playing a crucial role [Fig. 3 ▸(*e*)]. Utilizing these results, we predicted the full-length structure and substrate-binding mechanism of the MltG family.

*M. abscessus* is a pathogenic bacterium that causes serious infections, particularly in individuals with compromised immune systems or underlying lung conditions such as cystic fibrosis. The development of antibiotics specifically to target *M. abscessus* is crucial owing to its resistance to many conventional antibiotics. By elucidating the mechanisms and structural features involved in the transglycosyl­ation reaction catalyzed by MltG enzymes, our study deepens the understanding of bacterial cell wall biosynthesis. This knowledge can be leveraged to identify vulnerabilities in bacterial cell wall synthesis pathways that can be targeted by antibiotics. The catalytic details and implications of this structural plasticity between the putative PG-binding and catalytic domains will be an interesting research topic in the near future.

## Supplementary Material

PDB reference: MltG, 8yoa

## Figures and Tables

**Table 1:** Data collection and refinement statistics

Data collection	
X-ray source	PAL-5C
Wavelength (Å)	1.000
Space group	*P*2_1_2_1_2_1_
Unit-cell parameters	
*a*, *b*, *c* (Å)	43.22, 63.24, 123.56
α, β, γ (°)	90, 90, 90
Resolution range (Å)†	29.82–2.20 (2.34–2.20)
Total reflections	222278 (21829)
Unique reflections	17774 (1735)
Multiplicity	12.5 (12.6)
Completeness (%)†	99.37 (98.24)
Mean *I*/σ(*I*)†	16.51 (4.28)
*R*_merge_ (%)†‡	24.55 (91.07)
*R*_meas_ (%)†	3.9 (40.2)
CC_1/2_†	100 (96.7)
Wilson *B* factor (Å^2^)	30.24

Refinement	
Resolution range (Å)	29.82–2.2
Reflections (total/test set)	17774/889
*R*_work_ (%)	18.15
*R*_free_ (%)	21.93
No. of molecules in the asymmetric unit	1
No. of non-hydrogen atoms	2771
Macromolecules	2629
Solvent	142
Average *B* factor values (Å^2^)	33.30
Macromolecules	33.14
Solvent	36.30
Ramachandran plot: favored/allowed/outliers (%)	98.83/1.17/0.00
Rotamer outliers (%)	0.36
Clashscore	5.00
RMSD bonds (Å)/angles (°)	0.008/0.87

† Values for the outermost resolution shell in parentheses

‡ *R*_merge_ = Σ_h_Σ_*i*_|*I*(*h*)_*i*_ − 〈*I*(*h*)〉|/Σ*_h_*Σ*_i_I*(*h*)*_i_*, where *I*(*h*) is the observed intensity of reflection *h*, and 〈*I*(*h*)〉 is the average intensity obtained from multiple measurements.

**Table 2:** Structural similarity search using *DALI* PGD: PG-binding domain, CAD: catalytic domain.

Protein (PDB entry)	*Z* score	RMSD (Å)	RMSD (Å) b/w domains	Identity (%)
YceG-like protein from *L. monocytogenes* (4iiw)	24.7	9.8 (308/343)†	2.8 (PGD) 1.9 (CAD)	25
Amino­deoxy­chorismate lyase from *E. coli* (2r1f)	21.1	4.1 (259/343)†	2.8 (PGD) 4.2 (CAD)	22

† Number of residues aligned for RMSD calculation.

## Data Availability

Coordinates and structure factors have been deposited in the Protein Data Bank (PDB entry 8yoa).
